# Bis[2,6-bis­(2-meth­oxy­phen­yl)pyridinium] di-μ-bromido-bis­[dibromidocuprate(II)]

**DOI:** 10.1107/S1600536811003588

**Published:** 2011-02-05

**Authors:** Preeyanuch Sangtrirutnugul, Setsiri Haesuwannakij, Thanasat Sooksimuang, Samran Prabpai, Palangpon Kongsaeree

**Affiliations:** a Department of Chemistry and Center of Excellence for Innovation in Chemistry, Faculty of Science, Mahidol University, Rama VI Road, Bangkok 10400, Thailand; b National Metal and Materials Technology Center, Thailand Science Park, Klong Luang, Pathumthani, Thailand

## Abstract

The title salt, (C_19_H_18_NO_2_)_2_[Cu_2_Br_6_], was obtained from an attempt to synthesize the copper(II) complex of 2,6-bis­(2-meth­oxy­phen­yl)pyridine (*L*) from a reaction between CuBr_2_ and one equivalent of *L* in CH_2_Cl_2_ at room temperature. The resulting compound is the salt of the 2,6-bis­(2-meth­oxy­phen­yl)pyridinium cation and 0.5 equivalents of a hexa­bromido­dicuprate(II) dianion. Both meth­oxy groups of the cationic pyridinium moiety are directed towards the N atom of the pyridine ring as a result of intra­molecular N—H⋯O hydrogen bonds. The centrosymmetric hexabromidodicuprate dianion possesses a distorted tetra­hedral geometry at the copper ion. The Cu—Br bond lengths are 2.3385 (7) and 2.3304 (7) Å for the terminal bromides, whereas the bond length between the Cu atom and two bridging bromides is slightly longer [2.4451 (6) Å].

## Related literature

The neutral compound 2,6-bis­(2-meth­oxy­phen­yl)pyridine has been previously reported (Silva *et al.*, 1997 ▶) and copper(II) complexes of the related ligand 2,6-bis­(2′-hy­droxy­phen­yl)pyridine have also been characterized (Steinhauser *et al.*, 2004 ▶).
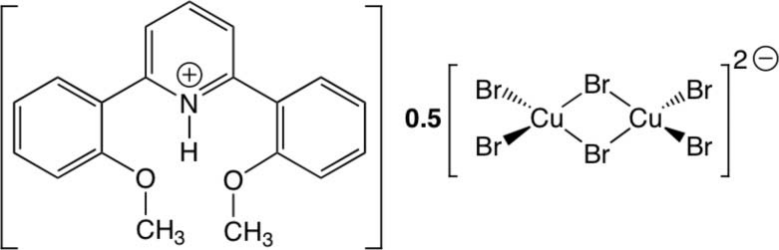


## Experimental

### 

#### Crystal data

(C_19_H_18_NO_2_)_2_[Cu_2_Br_6_]
*M* *_r_* = 1191.23
Orthorhombic, 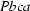

*a* = 11.5329 (1) Å
*b* = 17.0104 (4) Å
*c* = 21.0021 (5) Å
*V* = 4120.18 (14) Å^3^
*Z* = 4
Mo *K*α radiation
μ = 6.89 mm^−1^
*T* = 298 K
0.25 × 0.20 × 0.18 mm

#### Data collection

Nonius KappaCCD diffractometer
Absorption correction: multi-scan (*DENZO-SMN*; Otwinowski & Minor, 1997 ▶) *T*_min_ = 0.207, *T*_max_ = 0.301
28095 measured reflections
4177 independent reflections
3240 reflections with *I* > 2σ(*I*)
*R*_int_ = 0.075

#### Refinement

*R*[*F* ^2^ > 2σ(*F* ^2^)] = 0.042
*wR*(*F* ^2^) = 0.104
*S* = 1.05
4177 reflections
235 parameters
H-atom parameters constrained
Δρ_max_ = 0.36 e Å^−3^
Δρ_min_ = −0.55 e Å^−3^

### 

Data collection: *KappaCCD Server Software* (Nonius, 1997 ▶); cell refinement: *DENZO-SMN* (Otwinowski & Minor, 1997 ▶); data reduction: *DENZO-SMN*; program(s) used to solve structure: *SIR92* (Altomare *et al.*, 1994 ▶); program(s) used to refine structure: *SHELXL97* (Sheldrick, 2008 ▶); molecular graphics: *PLATON* (Spek, 2009 ▶); software used to prepare material for publication: *SHELXL97* and *maXus* (Mackay *et al.*, 1999 ▶).

## Supplementary Material

Crystal structure: contains datablocks global, I. DOI: 10.1107/S1600536811003588/vn2002sup1.cif

Structure factors: contains datablocks I. DOI: 10.1107/S1600536811003588/vn2002Isup2.hkl

Additional supplementary materials:  crystallographic information; 3D view; checkCIF report

## Figures and Tables

**Table 1:** Hydrogen-bond geometry (Å, °)

*D*—H⋯*A*	*D*—H	H⋯*A*	*D*⋯*A*	*D*—H⋯*A*
N1—H20⋯O1	0.96	1.90	2.625 (4)	131
N1—H20⋯O2	0.96	1.92	2.630 (4)	129

**Fig. 1.**
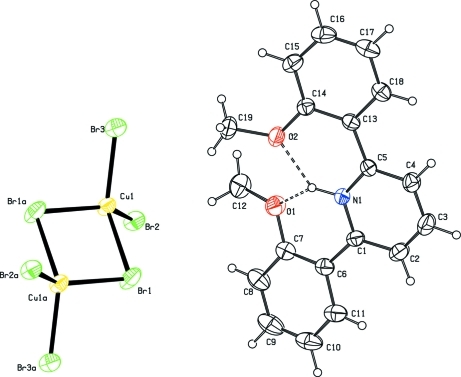
ORTEP diagram of the title compound (1). Displacement ellipsoids are drawn at the 30% probability level.

## supplementary crystallographic information

### Comment

An attempt to synthesize copper(II) complex of 2,6-bis(2-methoxyphenyl)- pyridine in CH_2_Cl_2_ unexpectedly yielded the ionic complex (C_9_H_18_NO_2_).0.5(Cu_2_Br_6_). The single crystals of the title compound crystallizes in the orthorhombic unit cell in space group P_bca_. Each asymmetric unit cell contains one molecule of 2,6-bis(2-methoxy- phenyl)pyridinium cation and half a molecule of hexabromodicuprate(II). Crystallographic data of the title compound reveals intramolecular N—H···O hydrogen bonds forcing both methoxy groups to be in close proximity to the nitrogen atom of the pyridinium ring (N···O distances of 2.625 (4) and 2.630 (4) Å). The pyridinium and two methoxyphenyl rings are almost co-planar, having the dihedral angles between them of 7.5 (5)° and 15.0 (5)°. In addition, weak intermolecular π-π stacking interactions between pyridine and phenyl moieties of the neighboring molecules with centroid-centroid distances of 3.649 (2) and 3.850 (2) Å are present.

Note that the centroid of the complete dianion coincides with the inversion center. Moreover, the hexabromodicuprate(II) dianion displays a distorted tetrahedral geometry at both copper(II) ions with Cu—Br bond distances of 2.3385 (7) and 2.3304 (7) Å for terminal bromides, and 2.4451 (6) Å for bridging bromides, respectively.

The neutral compound 2,6-bis(2-methoxyphenyl)pyridine has been previously reported (Silva *et al.*, 1997) and their crystals were obtained from an ethyl acetate solution. The published crystal structure reveals that both methoxy groups are on opposite sides of the pyridine nitrogen to avoid the N···O lone pair repulsion. In addition, copper(II) complexes of the related ligand 2,6-bis(2'- hydroxyphenyl)pyridine have previously been synthesized and characterized (Steinhauser *et al.*, 2004).

### Experimental

The title compound, (C_9_H_18_NO_2_).0.5(Cu_2_Br_6_) (1), was prepared from a reaction of CuBr_2_ (0.5 mmol) with one equivalent of 2,6-bis(2-methoxyphenyl)pyridine (0.5 mmol) in dichloromethane (30 ml) at room temperature for 3 h. The reaction solution was filtered to remove any unreacted CuBr_2_. X-ray quality single crystals were obtained from slow evaporation of a dichloromethane solution of 1 at room temperature.

### Refinement

Structure refinement was performed using least-squares analysis. All non-H atoms were refined anisotropically whereas all H atoms were placed in calculated positions and treated as riding with C,*N*—H = 0.96 with *U*_iso_(H) = 1.2 *U*_eq_(C,*N*), including the methoxy H atoms.

### Crystal data

**Table:**

(C_19_H_18_NO_2_)_2_[Cu_2_Br_6_]	*F*(000) = 2312
*M**_r_* = 1191.23	*D*_x_ = 1.920 Mg m^−^^3^
Orthorhombic, *P**b**c**a*	Mo *K*α radiation, λ = 0.71073 Å
Hall symbol: -P 2ac 2ab	Cell parameters from 32254 reflections
*a* = 11.5329 (1) Å	θ = 1.0–26.4°
*b* = 17.0104 (4) Å	µ = 6.89 mm^−^^1^
*c* = 21.0021 (5) Å	*T* = 298 K
*V* = 4120.18 (14) Å^3^	Cube, dark green
*Z* = 4	0.25 × 0.20 × 0.18 mm

### Data collection

**Table:**

Nonius KappaCCD diffractometer	3240 reflections with *I* > 2σ(*I*)
Radiation source: fine-focus sealed tube	*R*_int_ = 0.075
ω scans	θ_max_ = 26.4°, θ_min_ = 2.9°
Absorption correction: multi-scan (*DENZO-SMN*; Otwinowski & Minor, 1997)	*h* = −14→14
*T*_min_ = 0.207, *T*_max_ = 0.301	*k* = −21→21
28095 measured reflections	*l* = −22→26
4177 independent reflections	

### Refinement

**Table:**

Refinement on *F*^2^	Primary atom site location: structure-invariant direct methods
Least-squares matrix: full	Secondary atom site location: difference Fourier map
*R*[*F*^2^ > 2σ(*F*^2^)] = 0.042	Hydrogen site location: inferred from neighbouring sites
*wR*(*F*^2^) = 0.104	H-atom parameters constrained
*S* = 1.05	*w* = 1/[σ^2^(*F*_o_^2^) + (0.0575*P*)^2^ + 1.1145*P*] where *P* = (*F*_o_^2^ + 2*F*_c_^2^)/3
4177 reflections	(Δ/σ)_max_ < 0.001
235 parameters	Δρ_max_ = 0.36 e Å^−^^3^
0 restraints	Δρ_min_ = −0.55 e Å^−^^3^

### Special details

**Table:**

Experimental. multi-scan from symmetry-related measurements *SORTAV* (Blessing 1995)
Geometry. All standard uncertainties (except dihedral angles between l.s. planes) are estimated using the full covariance matrix. The standard uncertainties in cell dimensions are are used in calculating the standard uncertainties of bond distances, angles and torsion angles. Angles between l.s. planes have standard uncertainties calculated from atomic positional standard uncertainties; the errors in cell dimensions are not used in this case.

### Fractional atomic coordinates and isotropic or equivalent isotropic displacement parameters (Å^2^)

**Table:**

	*x*	*y*	*z*	*U*_iso_*/*U*_eq_	
Br1	−0.03538 (4)	0.45904 (3)	0.07154 (2)	0.07414 (19)	
Br2	0.14159 (4)	0.30603 (3)	0.00330 (2)	0.05795 (15)	
Br3	0.31781 (4)	0.47771 (3)	−0.02118 (2)	0.06266 (16)	
Cu1	0.12512 (4)	0.44281 (3)	−0.00337 (2)	0.05014 (16)	
N1	0.3781 (2)	0.23294 (16)	0.25843 (14)	0.0384 (7)	
H20	0.3754	0.2367	0.2128	0.046*	
O1	0.3184 (2)	0.16507 (18)	0.15107 (14)	0.0609 (8)	
O2	0.4577 (2)	0.30722 (17)	0.15766 (13)	0.0571 (7)	
C1	0.2958 (3)	0.1857 (2)	0.28504 (18)	0.0438 (9)	
C2	0.2983 (4)	0.1787 (3)	0.3505 (2)	0.0613 (11)	
H2	0.2431	0.1451	0.3713	0.074*	
C3	0.3783 (4)	0.2192 (3)	0.3857 (2)	0.0678 (12)	
H3	0.3768	0.2142	0.4312	0.081*	
C4	0.4595 (3)	0.2663 (2)	0.35705 (19)	0.0535 (10)	
H4	0.5156	0.2941	0.3822	0.064*	
C5	0.4602 (3)	0.2733 (2)	0.29171 (17)	0.0388 (8)	
C6	0.2118 (3)	0.1434 (2)	0.2446 (2)	0.0461 (9)	
C7	0.2227 (3)	0.1327 (2)	0.1792 (2)	0.0500 (10)	
C8	0.1406 (4)	0.0902 (3)	0.1446 (3)	0.0658 (12)	
H8	0.1496	0.0835	0.0995	0.079*	
C9	0.0476 (4)	0.0583 (3)	0.1753 (3)	0.0768 (16)	
H9	−0.0088	0.0294	0.1512	0.092*	
C10	0.0341 (4)	0.0672 (3)	0.2395 (3)	0.0751 (15)	
H10	−0.0311	0.0436	0.2606	0.090*	
C11	0.1135 (3)	0.1099 (3)	0.2742 (2)	0.0614 (12)	
H11	0.1027	0.1172	0.3191	0.074*	
C12	0.3434 (4)	0.1461 (3)	0.0865 (2)	0.0712 (13)	
H12A	0.4127	0.1730	0.0734	0.085*	
H12B	0.2798	0.1624	0.0600	0.085*	
H12C	0.3544	0.0904	0.0824	0.085*	
C13	0.5483 (3)	0.3218 (2)	0.25782 (18)	0.0421 (8)	
C14	0.5472 (3)	0.3374 (2)	0.19176 (19)	0.0445 (9)	
C15	0.6341 (4)	0.3824 (2)	0.1641 (2)	0.0569 (11)	
H15	0.6337	0.3919	0.1191	0.068*	
C16	0.7214 (4)	0.4135 (3)	0.2013 (3)	0.0671 (13)	
H16	0.7812	0.4448	0.1821	0.081*	
C17	0.7238 (3)	0.4001 (3)	0.2657 (3)	0.0669 (13)	
H17	0.7844	0.4222	0.2914	0.080*	
C18	0.6385 (3)	0.3547 (2)	0.2938 (2)	0.0550 (10)	
H18	0.6421	0.3458	0.3389	0.066*	
C19	0.4346 (4)	0.3398 (3)	0.0959 (2)	0.0667 (12)	
H19A	0.3696	0.3133	0.0772	0.080*	
H19B	0.5016	0.3331	0.0693	0.080*	
H19C	0.4174	0.3948	0.1000	0.080*	

### Atomic displacement parameters (Å^2^)

**Table:**

	*U*^11^	*U*^22^	*U*^33^	*U*^12^	*U*^13^	*U*^23^
Br1	0.0777 (3)	0.0785 (4)	0.0662 (3)	0.0310 (2)	0.0336 (2)	0.0342 (3)
Br2	0.0599 (3)	0.0497 (3)	0.0643 (3)	0.00570 (18)	0.01288 (19)	0.0059 (2)
Br3	0.0578 (3)	0.0655 (3)	0.0646 (3)	−0.00476 (19)	0.0102 (2)	0.0042 (2)
Cu1	0.0534 (3)	0.0490 (3)	0.0480 (3)	0.0075 (2)	0.0124 (2)	0.0064 (2)
N1	0.0394 (16)	0.0413 (16)	0.0344 (16)	0.0002 (12)	0.0000 (12)	0.0008 (13)
O1	0.0662 (18)	0.0702 (19)	0.0464 (18)	−0.0161 (15)	0.0017 (13)	−0.0131 (16)
O2	0.0643 (17)	0.0674 (19)	0.0395 (16)	−0.0155 (14)	−0.0049 (12)	0.0100 (14)
C1	0.0392 (19)	0.044 (2)	0.048 (2)	0.0010 (15)	0.0072 (16)	0.0039 (18)
C2	0.062 (3)	0.072 (3)	0.050 (3)	−0.013 (2)	0.011 (2)	0.007 (2)
C3	0.079 (3)	0.086 (3)	0.039 (2)	−0.009 (3)	0.003 (2)	0.004 (2)
C4	0.060 (2)	0.058 (3)	0.043 (2)	−0.0058 (19)	−0.0026 (18)	−0.005 (2)
C5	0.0388 (19)	0.0363 (19)	0.041 (2)	0.0044 (14)	−0.0021 (14)	−0.0026 (16)
C6	0.041 (2)	0.036 (2)	0.061 (3)	0.0040 (14)	0.0015 (17)	0.0058 (18)
C7	0.047 (2)	0.042 (2)	0.061 (3)	0.0006 (16)	−0.0013 (18)	−0.0037 (19)
C8	0.061 (3)	0.055 (3)	0.082 (4)	−0.004 (2)	−0.014 (2)	−0.014 (3)
C9	0.054 (3)	0.052 (3)	0.124 (5)	−0.006 (2)	−0.020 (3)	−0.012 (3)
C10	0.046 (3)	0.054 (3)	0.126 (5)	−0.0071 (19)	0.003 (3)	0.011 (3)
C11	0.045 (2)	0.054 (3)	0.085 (3)	0.0010 (19)	0.009 (2)	0.009 (2)
C12	0.089 (3)	0.071 (3)	0.054 (3)	0.001 (2)	0.002 (2)	−0.020 (2)
C13	0.0374 (18)	0.039 (2)	0.050 (2)	0.0028 (14)	−0.0009 (15)	−0.0031 (17)
C14	0.046 (2)	0.040 (2)	0.048 (2)	0.0004 (16)	0.0024 (16)	0.0020 (17)
C15	0.060 (3)	0.052 (3)	0.059 (3)	−0.001 (2)	0.014 (2)	0.010 (2)
C16	0.050 (3)	0.052 (3)	0.099 (4)	−0.008 (2)	0.012 (2)	0.008 (3)
C17	0.043 (2)	0.062 (3)	0.096 (4)	−0.006 (2)	−0.005 (2)	−0.007 (3)
C18	0.049 (2)	0.054 (2)	0.062 (3)	−0.0034 (18)	−0.0075 (19)	−0.003 (2)
C19	0.081 (3)	0.077 (3)	0.042 (3)	−0.005 (2)	−0.006 (2)	0.011 (2)

### Geometric parameters (Å, °)

**Table:**

Br2—Cu1	2.3385 (7)	C2—H2	0.9600
Br3—Cu1	2.3304 (7)	C19—H19A	0.9600
Br1—Cu1	2.4451 (6)	C19—H19B	0.9600
C5—N1	1.362 (4)	C19—H19C	0.9600
C5—C4	1.377 (5)	C3—H3	0.9600
C5—C13	1.490 (5)	C7—C6	1.392 (6)
O2—C14	1.356 (4)	C7—C8	1.396 (6)
O2—C19	1.435 (5)	C11—C6	1.413 (5)
O1—C7	1.367 (5)	C11—H11	0.9600
O1—C12	1.423 (5)	C12—H12A	0.9600
C14—C15	1.389 (5)	C12—H12B	0.9600
C14—C13	1.413 (5)	C12—H12C	0.9600
N1—C1	1.363 (4)	C13—C18	1.402 (5)
N1—H20	0.9600	C8—C9	1.364 (7)
C10—C9	1.365 (8)	C8—H8	0.9600
C10—C11	1.377 (7)	C18—C17	1.382 (6)
C10—H10	0.9601	C18—H18	0.9600
C4—C3	1.372 (6)	C16—C17	1.372 (7)
C4—H4	0.9600	C16—H16	0.9600
C15—C16	1.380 (6)	C17—H17	0.9600
C15—H15	0.9598	C9—H9	0.9600
C2—C3	1.368 (6)	C1—C6	1.476 (5)
C2—C1	1.380 (6)		
			
Br3—Cu1—Br2	100.69 (2)	O1—C7—C8	122.1 (4)
Br3—Cu1—Br1	142.79 (3)	C6—C7—C8	121.3 (4)
Br2—Cu1—Br1	97.77 (2)	C10—C11—C6	121.0 (5)
N1—C5—C4	117.6 (3)	C10—C11—H11	120.1
N1—C5—C13	120.5 (3)	C6—C11—H11	118.9
C4—C5—C13	121.8 (3)	O1—C12—H12A	109.4
C14—O2—C19	118.1 (3)	O1—C12—H12B	109.4
C7—O1—C12	118.9 (3)	H12A—C12—H12B	109.5
O2—C14—C15	122.5 (4)	O1—C12—H12C	109.6
O2—C14—C13	117.0 (3)	H12A—C12—H12C	109.5
C15—C14—C13	120.5 (4)	H12B—C12—H12C	109.5
C5—N1—C1	124.8 (3)	C18—C13—C14	117.4 (4)
C5—N1—H20	120.1	C18—C13—C5	118.0 (3)
C1—N1—H20	115.2	C14—C13—C5	124.5 (3)
C9—C10—C11	120.3 (5)	C9—C8—C7	119.6 (5)
C9—C10—H10	119.9	C9—C8—H8	120.4
C11—C10—H10	119.8	C7—C8—H8	120.1
C3—C4—C5	119.4 (4)	C17—C18—C13	121.4 (4)
C3—C4—H4	120.5	C17—C18—H18	118.5
C5—C4—H4	120.1	C13—C18—H18	120.0
C16—C15—C14	120.1 (4)	C17—C16—C15	120.6 (4)
C16—C15—H15	119.8	C17—C16—H16	119.5
C14—C15—H15	120.1	C15—C16—H16	119.9
C3—C2—C1	120.6 (4)	C16—C17—C18	120.0 (4)
C3—C2—H2	120.0	C16—C17—H17	120.2
C1—C2—H2	119.4	C18—C17—H17	119.9
O2—C19—H19A	109.5	C8—C9—C10	120.9 (5)
O2—C19—H19B	109.4	C8—C9—H9	119.1
H19A—C19—H19B	109.5	C10—C9—H9	120.0
O2—C19—H19C	109.5	N1—C1—C2	116.4 (3)
H19A—C19—H19C	109.5	N1—C1—C6	120.6 (3)
H19B—C19—H19C	109.5	C2—C1—C6	123.0 (3)
C2—C3—C4	121.2 (4)	C7—C6—C11	117.0 (4)
C2—C3—H3	118.9	C7—C6—C1	124.9 (3)
C4—C3—H3	120.0	C11—C6—C1	118.1 (4)
O1—C7—C6	116.5 (3)		

### Hydrogen-bond geometry (Å, °)

**Table:**

*D*—H···*A*	*D*—H	H···*A*	*D*···*A*	*D*—H···*A*
N1—H20···O1	0.96	1.90	2.625 (4)	131
N1—H20···O2	0.96	1.92	2.630 (4)	129
